# Prevalence and associated factors of perinatal asphyxia in newborns admitted to neonatal intensive care unit at the University of Gondar Comprehensive Specialized Hospital, Northwest Ethiopia, Ethiopia

**DOI:** 10.1186/s12887-021-03019-x

**Published:** 2021-11-27

**Authors:** Addisu Ginbu Dubie, Mehretie Kokeb, Abraham Tarkegn Mersha, Chilot Desta Agegnehu

**Affiliations:** 1 Department of pediatric and child health, School of Medicine, College of Medicine and health sciences, University of Gondar, Gondar, Ethiopia; 2 Department of Anesthesia and critical care, School of Medicine, College of Medicine and health sciences, University of Gondar, Gondar, Ethiopia; 3 School of Nursing, College of Medicine and Health Sciences and Comprehensive Specialized Hospital, University of Gondar, Gondar, Ethiopia

**Keywords:** Perinatal asphyxia, Newborn, Gondar, Ethiopia

## Abstract

**Background:**

Perinatal asphyxia is one of the leading causes of neonatal mortality and morbidity in Ethiopia. Understanding associated factors of perinatal asphyxia are important to identify vulnerable groups and to improve care during the perinatal period. Thus, this study aimed to assess the prevalence and associated factors of perinatal asphyxia among newborns admitted to NICU at the Gondar University Comprehensive Specialized Hospital Northwest Ethiopia, Ethiopia.

**Method:**

Institutional based cross-sectional study was conducted on 364 newborns from November 2018 - August 2019. Data was collected using a structured and pre-tested questionnaire. It was then cleaned, coded, and entered using EPI INFO version 7, then analyzed with SPSS statistics version 20.0. Binary logistic regression analysis was used to identify variables with p < 0.2. An adjusted odds ratio (AOR) with a 95% CI and *P-*value of <0.05 was used to identify significantly associated variables with perinatal asphyxia.

**Result:**

The prevalence of perinatal asphyxia in this study was 19.8, 95%CI (15.9, 24.2). Absence of maternal formal education (AOR = 4.09, 95%CI: 1.25, 13.38), pregnancy-induced hypertension (AOR = 4.07, 95%CI: 1.76, 9.40), antepartum hemorrhage (AOR = 6.35, 95%CI: 1.68, 23.97), prolonged duration of labor (AOR = 3.69, 95%CI: 1.68, 8.10), instrumental delivery (AOR = 3.17, 95%CI: 1.22, 8.21), and meconium-stained amniotic fluid (AOR = 4.50, 95%CI: 2.19, 9.26) were significantly associated with perinatal asphyxia.

**Conclusion:**

The prevalence rate of perinatal asphyxia in this study was comparable to other resource poor countries. The absence of maternal formal education, pregnancy-induced hypertension, and Antepartum hemorrhage, prolonged duration of labor, Instrumental assisted delivery, and meconium-stained amniotic fluid was having significant association with perinatal asphyxia in this study.

## Background

Globally, perinatal asphyxia is the most serious public health problem and occurs due to impaired blood gas exchange leading to progressive hypoxia [1].

According to World Health Organization (WHO), perinatal asphyxia is defined as “failure to initiate and sustain breathing at birth” [2]. APGAR score is one of the indicators used to describe the wellbeing of the newborn at birth [3]. It is difficult to detect the timing of birth asphyxia in different inevitability [4] that is the reason the severity of birth asphyxia is widely evaluated by the Apgar score, at 1 and 5 min next to birth [5]. Apgar score consists of five components as appearance (color), heart rate, grimaces (reflexes), activity (muscle tone), and respiration each of which is given a score of 0, 1, or 2. Apgar score < 7 for longer than 5 min indicated perinatal asphyxia [6–8].

Perinatal asphyxia is one of the leading causes of neonatal morbidity and mortality globally, especially in developing countries [9], and also the central cause of long–term diseases like cerebral palsy, mental retardation, irreversible neurologic damage, and epilepsy leading to detrimental long-term consequences for both the child and family [10–12].

Globally, every year more than 2.5 million infants die within the first month of life and nearly all deaths of newborns were in developing countries with the highest number of deaths were observed in Sub-Saharan Africa [13] In low-income countries 23% of all neonatal deaths occurred due to perinatal asphyxia [14]. In Africa, perinatal asphyxia is one of the top three causes of newborn deaths next to infection and prematurity [15, 16]. Various studies indicated the prevalence of perinatal asphyxia in Columbia was 41% [17], Nigeria 21% [16], Jimma southwest Ethiopia 12.5% [18], and in Dire Dawa, Ethiopia 2.5% [19]. Evidence indicated that the neonatal mortality rate is 29/1000 live births in Ethiopia and 23% and a large proportion of these neonatal death occur within the first 48 h after delivery [20].

Perinatal asphyxia can be affected by different factors. For example, obstetric complications [18], duration of labor [19, 21], maternal educational status, [19], lack of antenatal care, caesarian section, and weight of the fetus [21] can have an impact on the outcome of the newborn. Maternal, obstetrical, and fetal factors which result in decreased blood flow and oxygenation to the tissues can cause hypoxia in the fetus and asphyxia in the newborn [22]. Perinatal asphyxia is highly prevalent in developing countries as well as in Ethiopia. It is one of the top three causes of neonatal death. Therefore, this study aimed to investigate the prevalence of perinatal asphyxia and its associated factors among newborns admitted to the Neonatal Intensive Care Unit (NICU) at the University of Gondar Comprehensive Specialized Hospital Northwest Ethiopia, Ethiopia,

## Methods

### Study design and period

The institution-based cross-sectional study design was conducted at the University Gondar Comprehensive Specialized Hospital, from November 1st/2018 - August 31st/2019.

### Study area/setting

This study was conducted at the University of Gondar Comprehensive Specialized Hospital. It is located in Gondar city, Amhara National Region State, Northwest Ethiopia. It is the only comprehensive specialized teaching and referral hospital in the area with a total of 641 beds, 130 beds on the pediatrics side including the neonatal ward. It is the only hospital with NICU in the area.

NICU has 36 beds, 14 incubators, and 4 radiant warmers. The staff is composed of senior pediatricians, residents, interns, and BSc nurses. According to the hospital monthly mortality and morbidity report, there are about 150 neonatal admissions per month which give about 1800 admissions annually.

### Population and sample

The source population in this study was all new-born who were ever admitted to NICU at the University of Gondar Comprehensive Specialized Hospital and the study population was new-born who were admitted to NICU during the study period. The data was collected in this hospital from November 1st/2018 - August 31st/2019. By using systematic random sampling technique newborns with birth weight ≥ 1000 g or gestational age ≥ 28 weeks were included in the study.

The sample size was determined by using single population proportion formula assuming a 16.6% proportion from a previous study conducted at the University of Gondar referral hospital in 2014 [23], 95% confidence interval, and 4% marginal error.

$$n={\mathrm{Z}}^2\ast \mathrm{P}\ \left(1-\mathrm{p}\right)/{\mathrm{w}}^2$$

With Z of 1.96 at 95% confidence interval, the margin of error of 4%, and by adding a 10% non-response rate then the final sample size was 365.

### Variable of the study

The dependent variable was perinatal asphyxia, whereas the independent variables were socio-demographic characteristics (maternal age, marital status, educational status, and place of residence), antepartum factors (previous obstetrics history, ANC visits, obstetrics, and medical complications), Intrapartum factors (membrane rupture, duration of labor, obstructed labor, meconium-stained amniotic fluid, fetal presentation, mode and place of delivery), and fetal factors (Gestational age at birth, Apgar score, birth weight, sex of the newborn).

Newborns with birth weight ≥ 1000 g or gestational age ≥ 28 weeks were included in the study. Fifth minute Apgar score < 7 or requirement of positive pressure ventilation immediately after delivery with neurologic manifestations (hypo or hypertonia, irritability) was used to define perinatal asphyxia among the study participants [6–8]. Whereas, Newborns with major congenital anomalies incompatible with life such as hydrops, Neural tube defects, cyanotic congenital heart diseases, or chromosomal anomalies were excluded from the study. Newborns with opium or anesthesia-related low Apgar score were also excluded from this study.

### Data collection procedure and quality control

The data were collected by 4 intern doctors and 2 BSc nurses using a standardized pretested questionnaire. One day theoretical and practical training was given for the data collectors about the objective of the study. For each newborn, we have collected data regarding pregnancy, delivery, and neonatal admission characteristics. Socio-demographic data and medical history were taken by interview. Apgar score and diagnosis were filled by chart review. To ensure the quality of data, the questionnaire was pre-tested for consistency of understanding and completeness of data items on 10% of newborns at Obstetrics Labor Ward. The data collection process was closely monitored by the principal investigator throughout the data collection period. Completed questionnaires were checked regularly for completeness of the information and any gaps identified were immediately communicated to the data collectors.

### Data processing and analysis

Data were coded and entered into Epi info version 7.0 then exported to SPSS version 20 for cleaning and analysis. Summary statistics were carried out. Multicollinearity and model fitness was checked using Hosmer Lemeshow. Both Bi-variable and multivariable logistic regression analyses were used to identify associated factors of perinatal asphyxia. Variables with a *P-*value ≤ of 0.2 in the bi-variable logistic regression were entered into the multivariable logistic regression analysis. Adjusted odds ratio (AOR) with 95% confidence interval (CI) was computed. Variables with a *p-*value <0.05 in the multivariable logistic regression analysis were considered as significantly associated with perinatal asphyxia.

## Results

### Maternal socio-demographic characteristics

A total of 364 study participants were included in this study with a response rate of 99.7%. The majority of mothers 141(38.7%) were aged 25-29 years. Regarding marital status, 357(98.1%) of the mothers were married at the moment. Nearly one-third of the mothers (29.9%) had no formal education. Among the total study participants, 207(56.9%) were rural dwellers (Table 1**).**

**Table 1:** Sociodemographic characteristics of study participant mothers at the University Gondar Comprehensive Specialized Hospital, Northwest Ethiopia, November 2018 – August 2019 (*n* = 364)

Characteristics	Frequency (***n*** = 364)	Percentage (%)
**Age in years**		
15-19 years	18	4.9
20-24 years	131	36.0
25-29 years	141	38.7
30-34 years	47	12.9
≥ 35 years	27	7.4
**Marital status**		
Single	7	1.9
Married	357	98.1
**Educational status**		
No formal education	109	29.9
Primary	77	21.2
Secondary	104	28.6
Secondary+	74	20.3
**Residence**		
Urban	157	43.1
Rural	207	56.9

### Antepartum related characteristics

Among the study participants, 211(58%) were multiparous. About 346 (95.1%) mothers had a history of at least one ANC follow-up during the current pregnancy. Based on the previous obstetrics history of mothers 20 (5.5%) had an abortion, 18 (4.9%) had intrauterine death and 16(4.4%) had neonatal death. Twenty-one (8.5%) mothers had antepartum hemorrhage during pregnancy. In about 56(15.4%) mothers pregnancy-induced hypertension was diagnosed (Table 2).

**Table 2:** Ante-partum related characteristics of study participants at Gondar University Comprehensive Specialized Hospital, Northwest Ethiopia, November 2018 – August 2019 (*n* = 364)

Characteristics	Frequency (***n*** = 364)	Percentage (%)
**Parity**		
Primiparous	153	42
Multiparous	211	58
**ANC visit**		
Yes	346	95.1
No	18	4.9
**Previous delivery history**		
Abortion	20	5.5
Intrauterine death	18	4.9
Neonatal death	16	4.4
Perinatal asphyxia	5	1.4
**Medical complications**		
Pregnancy-induced hypertension	56	15.4
APH	21	5.8
Gestational DM/DM	7	1.9
Chorioamnionitis	6	1.6
Poly/oligohydramnios	14	3.8
Cardiac illness	1	0.3

### Intrapartum related characteristics

Out of the total participants, 336(92.3%) mothers had spontaneous onset of labor and in about 67(18.4%) mothers’ labor duration was more than 18 h. Nearly two-thirds (65.7%) of mothers had spontaneous vertex deliveries (SVD). In our study, inborn babies were more than outborn babies accounting for 257(70.6%) and 107(29.4%), respectively. In about 82(22.5%) mothers the amniotic fluid was meconium stained (Table 3).

**Table 3:** Intra-partum related characteristics of study participant at Gondar University Comprehensive Specialized Hospital, Northwest Ethiopia, November 2018 – August 2019 (*n* = 364)

Characteristics	Frequency (***n*** = 364)	Percentage (%)
**Time of membrane rupture**		
Premature	51	14
Intra-partal	313	86
**Type of labor**		
Spontaneous	336	92.3
Induced	28	7.7
**Duration of labor**		
< 18 h	297	81.6
≥ 18 h	67	18.4
**Mode of delivery**		
SVD	238	65.7
Instrumental	41	11.3
C/S	85	23.1
**Place of delivery**		
Inborn (At GHCSH)	257	70.6
Out born (Out of GUCSH)	107	29.4
**Fetal Presentation**		
Vertex	328	90.1
Nonvertex	36	9.9
**Obstructed labor**		
Yes	18	4.9
No	346	95.1
**Amniotic fluid**		
Stained	86	23.6
Non-stained	278	76.4
**Cord accident**		
Yes	2	0.5
No	362	99.5

### Neonatal related characteristics

Out of the total newborns, 213(58.5%) were male. Three-quarters (76.92%) of the newborn was a term. Three-fourth (73.1%) of the newborns had normal birth weight. There were 27(7.42%) twins among the study participants (Table 4).

**Table 4:** Neonatal related characteristics of study participant at Gondar University Comprehensive Specialized Hospital, Northwest Ethiopia, November 2018 – August 2019 (*n* = 364)

Characteristics	Frequency (***n*** = 364)	Percentage (%)
**Sex**		
Male	213	58.5
Female	151	41.5
**Gestational age**		
Preterm	61	16.75
Term	280	76.92
Post-term	23	6.32
**Birth weight**		
< 1500 g	18	4.9
1500-2499 g	67	18.4
2500-3999 g	266	73.1
≥ 4000 g	13	3.6
**Birth type**		
Single-tone	337	92.58
Twin	27	7.42

### Prevalence of perinatal asphyxia

The prevalence of perinatal asphyxia in newborns admitted to NICU at the University of Gondar Comprehensive Specialized Hospital was 19.8% with 95%CI (15.9, 24.2).

### Predictors of perinatal asphyxia

The bi-variable logistic regression analysis showed that maternal education, place of residence, parity, pregnancy-induced hypertension, antepartum hemorrhage, premature rupture of membrane, prolonged duration of labor, mode of delivery, place of delivery, fetal presentation, obstructed labor, meconium-stained amniotic fluid, gestational age, and birth weight were associated with perinatal asphyxia with *p-*value <0.2. However, the multivariable logistic regression analysis identified that absence of maternal formal education, pregnancy-induced hypertension, antepartum hemorrhage, duration of labor ≥18 h, instrumental delivery, and meconium-stained amniotic fluid were significantly associated with perinatal asphyxia.

The likelihood of developing perinatal asphyxia among neonates born from mothers who had not to attend formal education was 4 times (AOR = 4.09, 95%CI: 1.25, 13.38) higher compared with educated one. Neonates born from mothers with pregnancy-induced hypertension were 4.07 times (AOR = 4.07, 95%CI: 1.76, 9.40) more likely to develop perinatal asphyxia compared with their counterparts. Neonates born from mothers with Antepartum Hemorrhage had 6.4 times (AOR = 6.35, 95%CI: 1.68, 23.97) higher risk of developing perinatal asphyxia as compared to their counterparts. Mothers who had prolonged labor were 3.7 times (AOR = 3.69, 95%CI: 1.68, 8.10) more likely to have asphyxiated newborns than those who had a normal duration of labor. Regarding the mode of delivery, those newborns born through instrumental assisted were 3.2 times (AOR = 3.17, 95%CI: 1.22, 8.21) more likely to develop perinatal asphyxia than newborns delivered through spontaneous vaginal delivery. Neonates born with meconium-stained amniotic fluid were 4.5 times (AOR = 4.50, 95%CI: 2.19, 9.26) as likely to have perinatal asphyxia (Table 5**).**

**Table 5:** Bi variable and multivariable logistic regression analysis of associated factors of perinatal asphyxia, University of Gondar Comprehensive Specialized Hospital, Northwest Ethiopia, November 2018 – August 2019 (*n* = 364)

Variables	Perinatal asphyxia		COR (95%CI)	AOR (95%CI)	*P-*value
	Yes	No			
**Educational status**					
No education	39	70	5.33 (2.23, 12.75)	4.09 (1.25, 13.38)	0.02
Primary	11	66	1.60 (0.58, 4.37)	0.98 (0.27, 3.48)	0.97
Secondary	15	89	1.61 (0.62, 4.18)	1.49 (0.47, 4.73)	0.49
Secondary and above	7	67	1	1	
**Place of residence**					
Urban	12	137	1	1	
Rural	52	155	0.44 (0.25, 0.77)	0.79 (0.32, 1.93)	0.6
**Parity**					
Primiparous	38	115	1.72 (1.02, 2.89)	1.21 (0.59, 2.46)	0.6
Multiparous	34	177	1	1	
**Pregnancy-induced HTN**					
Yes	19	37	2.47 (1.32, 4.63)	4.07 (1.76, 9.40)	0.001
No	53	255	1	1	
**APH**					
Yes	7	14	2.14 (0.83, 5.51)	6.35 (1.68, 23.97)	0.006
No	65	278	1	1	
**Membrane rupture**					
Premature	19	32	2.91 (1.54, 5.52)	2.06 (0.88, 4.83)	0.09
Intra-partal	53	260	1	1	
**Duration of labor**					
≥ 18 h	33	34	6.42 (3.58, 11.53)	3.69 (1.68, 8.10)	0.001
< 18 h	39	258	1	1	
**Place of delivery**					
Inborn (At GUCSH)	46	211	1	1	
Out born (Out of GUCSH)	26	81	0.68 (0.39, 1.17)	0.87 (0.40, 1.90)	0.73
**Mode of delivery**					
SVD	45	193	1	1	
Instrumental	15	26	1.42 (0.71, 2.83)	3.17 (1.22, 8.21)	0.018
C/S	12	73	3.51 (1.45, 8.47)	3.33 (1.00, 11.09)	0.05
**Fetal presentation**					
Vertex	60	268	1	1	
Non-vertex	12	24	0.45 (0.21, 0.95)	0.47 (0.16, 1.38)	0.16
**Obstructed labor**					
Yes	11	7	7.34 (2.74, 19.70)	1.88 (0.50, 7.01)	.347
No	61	285	1	1	
**Amniotic fluid**					
Stained	36	50	4.84 (2.78, 8.42)	4.50 (2.19, 9.9.26)	0.001
Non-stained	36	242	1	1	
**Gestational age**					
Pre term	7	54	0.49 (0.21, 1.12)	0.56 (0.10, 3.22)	0.51
Term	59	221	1	1	
Post term	6	17	1.32 (0.50, 3.50)	0.69 (0.21, 2.32)	0.54
**Birth weight**					
< 1500 g	1	17	0.22 (0.03, 1.66)	0.31 (0.02, 5.93)	.437
1500-2499 g	9	58	0.57 (0.27, 1.22))	0.80 (0.20, 3.25)	.758
2500-3999 g	57	209	1	1	
≥ 4000 g	5	8	2.29 (0.72, 7.28)	1.18 (0.28, 4.95)	.819

*COR* Crude Odds Ratio, *AOR* Adjusted Odds Ratio

## Discussion

Perinatal asphyxia is one of the major causes of neonatal death during the neonatal period. Therefore, this study aimed to assess the prevalence and associated factors of perinatal asphyxia in newborns admitted to NICU at the University of Gondar Comprehensive Specialized Hospital with level III NICU care.

In this study, the prevalence of perinatal asphyxia was found to be 19.8% (15.9, 24.2). This study is in line with other studies done in Nigeria (21.1%) [16], Tigray Ayder hospital with level IV NICU care (18%) [21], and higher than the previous report from the University of Gondar Referral Hospital in 2013 (13.8%) [24]. This might be due to increased referral cases from time to time in different primary hospitals and health centers either after detecting obstetrics complications or attending deliveries of asphyxiated babies because the University of Gondar Comprehensive Specialized Hospital is the only specialized hospital in the catchment area with Neonatal Intensive Care Unit. On the other hand, the prevalence rate obtained in this study was lower than what has been observed in Dilla University Referral Hospital with level III NICU care (32.8%) [25]. In Dilla, low birth weight was a significant risk factor of perinatal asphyxia and there was a high proportion of low birth weight in Dilla as compared to Gondar, this may increase the prevalence of perinatal asphyxia.

In this study, there was a statistically significant association between perinatal asphyxia and maternal educational status. Neonates born from mothers who had not attend formal education were four times more likely to develop perinatal asphyxia. This finding is consistent with studies done in Ethiopia in Dilchora Referral Hospital with level II NICU care, Kenya kaka mega county referral hospital, Ghana, Tanzania, and Pakistan [19, 26–29]. This may be due to women without formal education might have a lack of awareness about maternal health care services like antenatal care visits. Maternal illiteracy is a very broad indicator of poor socio-economic conditions associated with malnutrition, frequent pregnancies, and delay in care-seeking during the antepartum and intrapartum period.

Pregnancy-induced hypertension was also observed as a significant risk factor of perinatal asphyxia in our study. Neonates born from mothers with pregnancy-induced hypertension were four times more likely to develop perinatal asphyxia compared with their counterparts. This study is consistent with studies conducted in Jimma, India, Cameroon, and Pakistan [6, 18, 30, 31]. Pregnancy-induced hypertension can result in a decrease in placental blood flow and loss of placental integrity which can lead to an inadequate fetoplacental blood flow causing intrauterine growth retardation and perinatal asphyxia [32].

Prolonged duration of labor was another risk factor of perinatal asphyxia. Newborns delivered from mothers who spent more than 18 h in labor were 3.7 times more likely to develop perinatal asphyxia as compared to their counterparts. This result is consistent with previous studies done at the University of Gondar Referral Hospital [24], in Dilchora referral Hospital [19], Cameroon, Kenya, Nigeria, and Sweden [6, 16, 26, 33]. Prolonged labor is likely to occur if the women have a narrow pelvis, poor uterine contraction, or slow cervical effacement. If the labor does not progress normally, there may be serious complications such as uterine rupture, hemorrhage, maternal infection, neonatal infection, and fetal distress. All of these complications can lead to perinatal asphyxia. Furthermore, the child experience prolonged or arrested labor may start to experience birth asphyxia due to umbilical cord problems or the stress of too many contractions [34].

Antepartum hemorrhage is another risk factor of perinatal asphyxia. Newborns delivered from mothers with antepartum hemorrhage were 6.4 times more likely to develop perinatal asphyxia. This finding is similar to a study conducted in Pakistan and Indonesia [19, 24]. During antepartum bleeding, there will be decreased blood flow from the mother to the fetus which can lead to perinatal asphyxia if maternal transfusion or delivery is delayed.

Regarding the mode of delivery, newborns delivered with instrumental assisted delivery were 3.2 times more likely to develop perinatal asphyxia. This finding is similar to a study conducted in Dilchora referral Hospital, Ethiopia, and India [19, 30]. This finding might be because either most of the mothers came with possible complications or the decision to assist with the instrument might be delayed till they develop a complication. Most of the indications for instrumental delivery can compromise adequate oxygen supply to the fetus, which might result in fetal distress and perinatal asphyxia. In general in developing countries including Ethiopia mothers and neonates were prone to morbidity and mortality due to instrumental delivery [35].

Neonates born from mothers with a history of meconium-stained amniotic fluid were four and half times more likely to develop perinatal asphyxia. This is consistent with previous studies conducted at the University of Gondar Referral Hospital [24], Jimma public Hospitals [18], Cameroon [30], Pakistan [31], India [6], and Nigeria [16]. This might be explained by meconium aspiration syndrome leading to airway obstruction and subsequent hypoxia. The possible reason could be intrapartum inhalation of meconium-stained amniotic fluid resulted in chemical pneumonitis with the inflammation of pulmonary tissues, the aspiration syndrome leading to airway obstruction, and pulmonary air leak, this in turn to hypoxia that is prenatal asphyxia [36].

Even though, there is a standard guideline for neonatal resuscitation; the death of neonatal mortality due to perinatal asphyxia is increasing in Ethiopia. This means perinatal asphyxia is a common problem in our country so every stakeholder better use different strategies to reduce the burden of perinatal asphyxia by taking the appropriate action on the determinants.

The limitation of this study was some potential predictors for the low Apgar score were not considered like placental factors, maternal anemia, and intrauterine infections.

## Conclusion

The prevalence rate of perinatal asphyxia in this study was comparable to other poor rescores countries. The absence of maternal education, pregnancy-induced hypertension, antepartum hemorrhage, prolonged duration of labor, instrumental assisted delivery, and meconium-stained amniotic fluid was found to be predictors of perinatal asphyxia. Therefore, to reduce the burden of perinatal asphyxia better to improve the quality of intrapartum care by implementing different strategies to prevent prolonged labor, identify obstetrics complications, ascertain and make a strict follow up of mothers with meconium-stained amniotic fluid. Addressing and identifying determinants of perinatal asphyxia may improve the application of the WHO standard guideline effectively and consistently.

## Data Availability

The datasets generated and/or analyzed during the current study are not publicly available due to confidentiality but are available from the corresponding author on reasonable request.

## Abbreviations

ANC: Ante-Natal Care
APGAR: Appearance, Pulse rate, Grimace, Activity, Respiration rate
C/S: Cesarean section
GA: Gestational age
UOGCSH: University of Gondar Comprehensive Specialized Hospital
NICU: Neonatal Intensive Care Unit
COR: Crude odds ratio
AOR: Adjusted odds ratio
PNA: Perinatal Asphyxia
PROM: Premature Rupture of Membrane
SPSS: Statistical Package for Social Science
SVD: Spontaneous Vertex delivery
WHO: World Health Organization
